# An Investigation of the Factors Affecting the Length of Hospitalization of Diabetic Foot Patients Who Underwent Minor Amputation

**DOI:** 10.7759/cureus.79672

**Published:** 2025-02-26

**Authors:** Shinsuke Imaoka, Genki Kudou, Shohei Minata

**Affiliations:** 1 Rehabilitation Department, Oita Oka Hospital, Oita, JPN

**Keywords:** diabetes, diabetic foot, length of hospital stay, minor amputation, rehabilitation

## Abstract

Background

While progress in multidisciplinary therapies has substantially improved the limb-sparing rate of patients with diabetic foot or peripheral artery disorders, the requirement for multiple resting periods for the recovery of foot disorders has resulted in prolonged hospitalization. This study aimed to determine the factors that significantly affect the length of hospital stay in patients with diabetic foot who underwent minor amputation.

Methodology

This study included 95 patients with diabetic foot who underwent minor amputation followed by rehabilitation between April 2013 and June 2016. We retrospectively evaluated the factors available in the medical records. The factors included age, sex, body mass index, level of amputation, presence of hemodialysis, preoperative weight bearing index, motor ability, discharge destination, load reduction period, Barthel index, Hasegawa Dementia Scale-Revised (HDS-R), preoperative blood tests (C-reactive protein and serum albumin), and average number of units of rehabilitation. Spearman’s rank correlation coefficient and multiple regression analysis were used for statistical analysis.

Results

Multiple factors, including load reduction period, HDS-R, average number of units of rehabilitation provided, and the level of amputation, were significantly correlated with the duration of hospital stay.

Conclusions

Shortening the load reduction period and intensive rehabilitation at an early stage after amputation should be prioritized to minimize hospitalization in patients with diabetic feet.

## Introduction

Diabetic foot lesions are infections, ulcers, and deep tissue destruction of the lower extremity with neuropathy and peripheral blood flow impairment [1]. As in the United States, the number of non-traumatic amputees is rapidly increasing, with peripheral vascular disorders accounting for 49.0% and diabetes mellitus for 28.6% of all amputations in a Japanese survey of causes [2]. In Japan, with its aging population, the increase in the number of lower limb amputees is directly linked to the increase in the number of bedridden patients and is a factor in increasing medical and social security costs. Therefore, since 2000, multidisciplinary treatment for diabetic foot lesions and peripheral artery disease has progressed, and lower limb preservation treatment that avoids major amputation and preserves lower limb function has become widespread, reducing the number of patients with major amputations [3-5]. On the other hand, patients with minor amputations who have preserved lower limb function have a poor healing rate and a high rate of plantar ulceration and reamputation [6,7]. In addition, studies on length of hospital stay have shown that patients with minor amputations tend to have longer hospital stays than those treated conservatively [8]. This is because a period of offloading is required after a minor amputation, especially in the elderly, which may delay recovery of mobility and activities of daily living (ADLs) and increase the length of hospital stay. Therefore, the effectiveness of early rehabilitation in improving postoperative gait function and ADL has recently been reported in patients with diabetic foot disease [9,10]. However, previous studies on the length of hospitalization of diabetic foot patients undergoing rehabilitation after minor amputation are scattered and have not been validated in detail [11]. Against this background, we hypothesized that identifying factors that influence the length of hospitalization of patients undergoing rehabilitation after minor amputation for the treatment of diabetic foot lesions could inform rehabilitation intervention strategies. Therefore, the purpose of this study was to identify factors that influence the length of hospitalization of patients who underwent rehabilitation after a minor amputation for the treatment of diabetic foot lesions.

## Materials and methods

**Figure 1:**
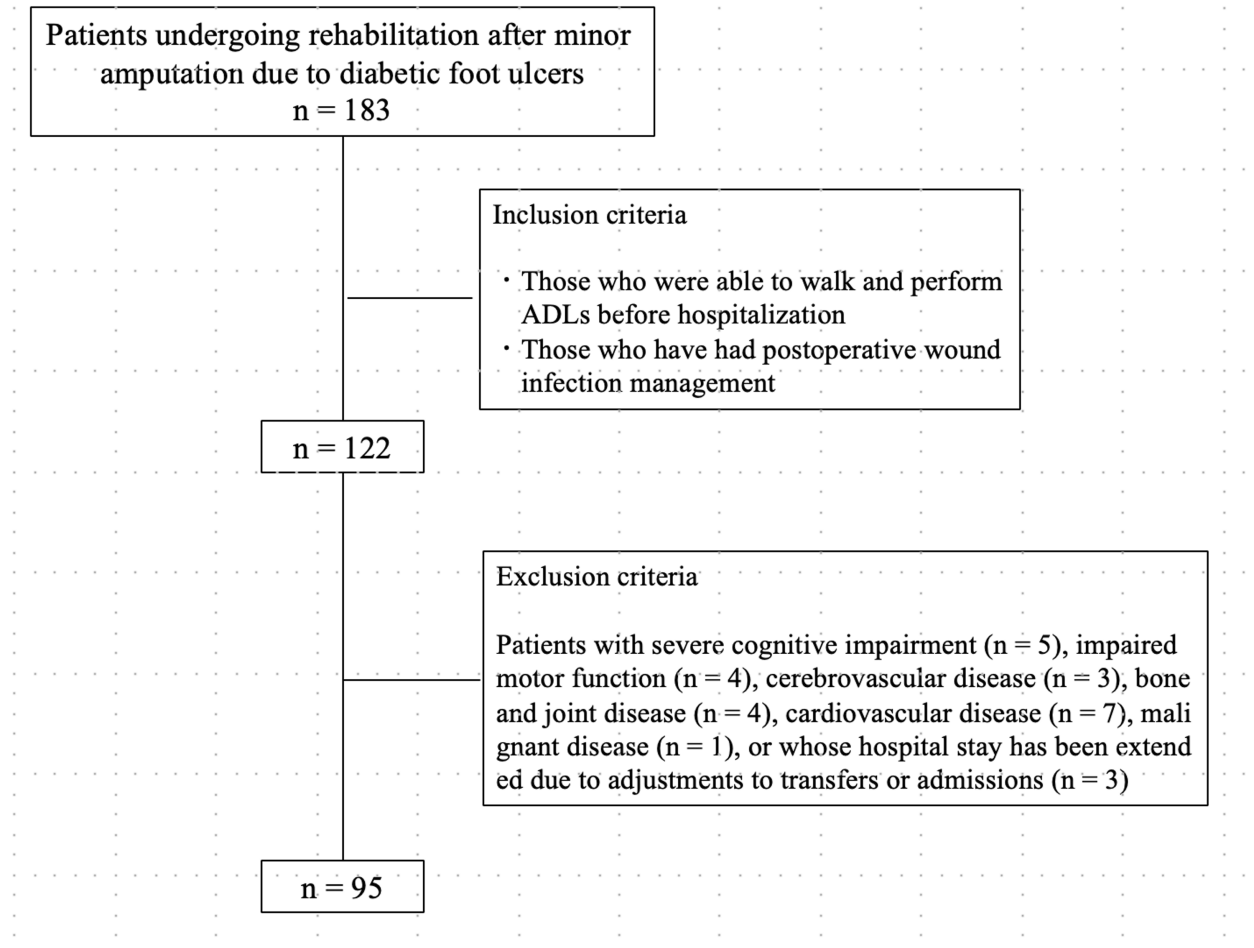
Flowchart of patient selection. ADLs: activities of daily living

This was a retrospective, observational study. A total of 158 patients who were admitted to the plastic surgery ward of our hospital between April 2013 and March 2016 with diabetic foot ulcers underwent minor amputation and rehabilitation. Of the 122 patients who met the inclusion criteria, 95 were analyzed after excluding those who met the exclusion criteria (52 men and 43 women). The mean patient age was 72.8 ± 15.3 years (Figure 1).

The inclusion criteria were minor amputation for limb salvage, the ability to walk and perform ADLs before admission, and postoperative wound infection control. The exclusion criteria were a history of cerebrovascular disease causing significant cognitive or motor impairment, bone and joint disease, cardiovascular disease, malignant disease, and extended hospital stay due to transfer arrangements or social background factors. Survey items included age, sex, number of hospitalization days, body mass index (BMI), and schizophrenia. The following items were retrospectively surveyed from medical records: BMI, offloading period, whether or not artificial dialysis was performed, amputation level (toe amputation, multiple toe amputation, trans-metatarsal amputation, Lisfranc amputation), whether or not wound healing occurred, time to wound healing, preoperative blood test results (serum albumin, C-reactive protein), Barthel Index at admission and discharge, Hasegawa Dementia Scale-Revised (HDS-R), mobility at admission and discharge (functional walking or wheelchair), preoperative Weight-Bearing Index (WBI), average number of rehabilitation units provided, and discharge destination (home or transfer). The offloading period was the period from after minor amputation to the start of weight-bearing on the operated side. The conditions for starting weight-bearing were that bleeding and pain could be controlled, and pressure around the operated area could be decompressed. The average number of rehabilitation units provided was calculated by dividing the total number of units acquired by the number of days of hospitalization. For mobility, patients who could walk in the ward, excluding stairs, with the assistance of family or ward staff were classified as the practical walking group. Patients who were discharged to their home, group home, paid nursing home, or senior housing with services were classified as the home group. The main rehabilitation interventions during the bed rest period were (1) lower limb muscle strengthening exercises (knee extension and hip abduction) and (2) triceps surae stretching to maintain ankle dorsiflexion mobility while protecting the operated area. After starting weight-bearing, standing and walking exercises were performed using compression sandals. The purpose of rehabilitation intervention in patients with minor amputation is to maintain walking ability and ADLs, and the goal is wound healing and the acquisition of practical walking ability at the time of discharge.

Statistical analysis was performed by testing the correlation coefficient between the length of hospital stay and each survey item using Spearman’s rank correlation coefficient. In addition, multiple regression analysis was performed using the stepwise method, with the length of hospital stay as the dependent variable and items that were correlated with the length of hospital stay as the independent variables. To consider multicollinearity, we confirmed whether there was a strong correlation between the independent variables. Before these tests, the normality of all data was confirmed using the Shapiro-Wilk test. Statistical analysis was performed using SPSS Statistics version 22.0 (IBM Corp., Armonk, NY, USA), and the significance level was set at 5%.

This study was approved by the Ethics Committee of the Oita Oka Hospital (approval number: A056). Information about the study was made publicly available on the Keiwakai Oita Oka Hospital website, and a means was provided for subjects and their families to withdraw from participation in the study.

## Results

Patient background and evaluation indices

The average age of the patients was 72.8 ± 15.3 years. Overall, there were 52 men (53.6%) and 43 women (46.4%). The BMI was 23.3 ± 7.7 kg/m^2^, and the mean length of hospital stay was 43.1 ± 11.7 days. Amputation levels included 33 (34.7%) toe amputations, 28 (29.4%) multiple toe amputations, 23 (24.1%) trans-metatarsal amputations, and 11 (11.5%) Lisfranc disarticulations. The progress in mobility was 28.4% walking independently at the time of admission (18.9% at discharge), 32.6% walking independently with a cane (37.8% at discharge), 20.0% assisted with a cane (21.0% at discharge), and 18.9% wheelchair users (22.1% at discharge). Wound healing was achieved in 87 (91.5%) patients, and the time to wound healing was 31.2 ± 14.6 days (Table 1).

**Table 1:** Patient attributes (n = 95). Mean ± standard deviation. Alb: serum albumin level; CRP: C-reactive protein; CRP: C-reactive protein; BI: Barthel Index; HDS-R: Hasegawa Dementia Scale-Revised; WBI: Weight-Bearing Index

Variables	All
Age (years)	72.8 ± 15.3
Gender (n)	Male = 52
	Female = 43
Hospital stays (days)	43.1 ± 11.7
BMI (kg/m^2^)	23.3 ± 7.7
Offloading period (days)	13.7 ± 6.8
Dialysis (n)	32
Amputation level (limb)	Toe = 33
	Multiple toe = 28
	Trans-metatarsal = 23
	Lisfranc = 11
Alb (g/dL）	3.5 ± 1.5
CRP (mg/dL)	0.59 ± 0.24
BI at admission (points)	77.5 ± 17.8
BI at discharge (points)	69.8 ± 21.8
HDS-R (points)	25.9 ± 4.8
Mobility at admission (n)	Walking independently = 27
	With a cane, independence = 31
	With a cane, assistance = 19
	Wheelchair = 18
Mobility at discharge (n)	Walking independently = 18
	With a cane, independence = 36
	With a cane, assistance = 20
	Wheelchair = 21
Preoperative WBI (%BW)	0.29 ± 0.18
Average number of rehabilitation units provided (points)	2.8 ± 1.7
Wound healing (n)	87
Time to wound healing (days)	31.2 ± 14.6
Discharged home (n)	72

Correlation coefficient between the length of hospital stay and each evaluation index

A moderately significant correlation was confirmed between the length of hospital stay and age (r = 0.53), amputation level (r = 0.44), preoperative WBI (r = -0.45), unweighted period (r = -0.71), HDS-R (r = -0.51), and the average number of rehabilitation units provided (r = -0.40) (p < 0.05) (Table 2).

**Table 2:** Correlation between length of hospitalization and each item. Alb: serum albumin level; CRP: C-reactive protein; BI: Barthel Index; HDS-R: Hasegawa Dementia Scale-Revised; WBI: Weight-Bearing Index

	Correlation coefficient (r)	P-value
Age (years)	0.58	0.01
Gender (n)	0.09	0.34
BMI (kg/m^2^)	0.15	0.28
Offloading period (days)	-0.74	0.01
Dialysis (n)	0.13	0.23
Amputation levels (limb)	0.53	0.01
Alb (g/dL）	0.15	0.15
CRP (mg/dL)	0.12	0.23
BI at admission (points)	-0.02	0.68
BI at discharge (points)	0.26	0.48
HDS-R (points)	-0.51	0.01
Mobility on admission (n)	-0.15	0.12
Mobility at discharge (n)	-0.32	0.09
Preoperative WBI (%BW)	-0.49	0.01
Average number of rehabilitation units provided (points)	-0.42	0.01
Wound healing, yes (n)	0.18	0.39
Time to wound healing (days)	-0.46	0.08
Discharged home (n)	0.22	0.43

Multiple regression analysis with hospital days as the dependent variable

In the multiple regression analysis with hospital days as the dependent variable, the following independent variables were extracted as influential factors: unweighting period (β = 0.54), average number of rehabilitation units provided (β = -0.20), HDS-R (β = -0.28), and amputation level (β = -0.24). The results of the analysis of variance were significant (p < 0.01), and the coefficient of determination R^2^ was 0.51 (Table 3).

**Table 3:** Factors determining the length of stay. Analysis of variance, p < 0.01; adjusted R^2^ = 0.51. HDS-R: Hasegawa Dementia Scale-Revised

Variable	Partial regression coefficient	Standard regression	P-value
Offloading period	1.211	0.545	0.01
Average number of rehabilitation units provided	-4.686	-0.206	0.01
HDS-R	-0.681	-0.288	0.01
Amputation level	5.284	0.246	0.01

## Discussion

In this study, the offloading period, average number of rehabilitation units provided, HDS-R score, and amputation level were extracted as factors influencing hospital stay. It is generally known that long-term immobilization and unloading after tissue damage or surgery can cause bone loss and muscle atrophy, leading to decreased mobility and delayed social reintegration [12,13].

In addition, many reports have been published on the relationship between diabetes and lower limb muscle weakness [14,15]. It has been predicted that the loss of physical function during the postoperative rest period is more significant in unhealthy older adults than in healthy ones. Regarding the relationship between the offloading period and the number of days of hospitalization, it has been shown that an average of six to eight weeks of unloading is required for the healing of diabetic foot lesions [16].

As most of the patients in this study were older adults and had diabetes, it is possible that the rapid decline in physical and mental functions during the offloading period and the time it took to regain mobility may have influenced the number of days of hospitalization. Regarding the relationship between the amount of rehabilitation provided and length of hospitalization, surveys at acute care medical institutions revealed that the more frequent the rehabilitation intervention, the shorter the length of hospitalization, the higher the possibility of returning home, and the better the improvement in ADL ability [17,18].

Although there are differences in the target diseases and medical management methods, this factor is expected to play an important role in preventing disease progression in older patients. Regarding the relationship between cognitive decline and hospitalization period, it has been shown that patients with cognitive decline have a higher in-hospital mortality rate and incidence of delirium, and the length of hospitalization is longer [19]. In patients with diabetes who underwent minor amputations, many are required to follow a bed rest regime and wear specialized footwear early after surgery.

It is possible that cognitive decline interferes with bed rest management, delays wound healing, and prolongs the time needed to adhere to footwear regulations, which may affect the length of hospitalization. In addition, it has been reported that a higher amputation level is associated with more difficulty in regaining the ability to walk and a poorer prognosis [20]. On the other hand, patients with multiple toe and trans-metatarsal amputations who were able to walk tended to be discharged later due to the need for interventions focused on preventing recurrence and adjusting footwear. This observation requires more detailed verification and intervention. The clinical significance of this study is underscored by the expected continued increase in the proportion of diabetic patients undergoing minor amputations as multidisciplinary medical care aimed at limb salvage is promoted.

Finally, the limitations and future challenges of this study include the retrospective observational design using medical records from a single facility, a small sample size for verification, and insufficient standardization of the rehabilitation interventions. In addition, selection bias may have occurred because the inclusion criterion required patients to be capable of performing ADLs, such as walking, before hospitalization, and these factors could not be fully controlled.

## Conclusions

In this study, the following factors were extracted: offloading period, average number of units provided, HDS-R, and amputation level. Among the extracted factors, HDS-R is heavily influenced by the condition before hospitalization, and amputation level is difficult to intervene in because it is determined by the level of the lesion. However, the non-weight-bearing period and the average number of units provided in rehabilitation may change after surgery, and interventions in these areas may shorten the length of hospital stay.
